# Design of Strain-Hardening Natural TRM Composites: Current Challenges and Future Research Paths

**DOI:** 10.3390/ma16134558

**Published:** 2023-06-24

**Authors:** Rogiros Illampas, Daniel V. Oliveira, Paulo B. Lourenço

**Affiliations:** University of Minho, Institute for Sustainability and Innovation in Structural Engineering, Associate Laboratory Advanced Production and Intelligent Systems, Department of Civil Engineering, 4800-058 Guimarães, Portugal; danvco@civil.uminho.pt (D.V.O.); pbl@civil.uminho.pt (P.B.L.)

**Keywords:** natural fibers, textile-reinforced mortar (TRM), composite materials, tensile response

## Abstract

This paper discusses the challenges in using natural fibers for the development of textile-reinforced mortar (TRM) composites with pseudo-strain-hardening and multiple cracking behavior. The particular characteristics of natural vegetal fibers are analyzed with reference to data from the literature. It is concluded that the efficient use of these fibers as composite reinforcement requires the development of treatment or impregnation protocols for overcoming durability issues, eliminating crimping effects in tensile response and imparting dimensional stability. Relevant experimental research on the synthesis and performance of natural TRMs is reviewed, showing that the fabrication of such systems is, at present, largely based on empirical rather than engineering design. In order to set a framework regarding the properties that the constituents of natural TRM must meet, a comparative analysis is performed against inorganic matrix composites comprising synthetic, mineral and metallic reinforcement. This highlights the need for selecting matrix materials compatible with natural fibers in terms of stiffness and strength. Furthermore, a rational methodology for the theoretical design of natural TRM composites is proposed. First-order analysis tools based on rule-of-mixtures and fracture mechanics concepts are considered. Based on the findings of this study, paths for future research are discussed.

## 1. Introduction

Textile-reinforced mortars (TRMs) are composite materials consisting of fiber textiles embedded in inorganic matrices (most commonly cement- or lime-based mortars). Such composites can be used as externally bonded reinforcement in the strengthening of existing buildings [1,2] or as structural materials for the construction of free-form structures and prefabricated components [3,4]. More recently, they have also been combined with thermal retrofitting systems for the concurrent structural and energy upgrading of buildings [5,6,7]. TRM systems offer several benefits, including a high strength-to-weight ratio, ease of application and minimal change in member geometry when used as strengthening layers. However, key challenges with respect to the sustainability and cost-efficiency of TRM solutions still need to be answered. The synthetic and mineral fiber textiles (e.g., glass, basalt, carbon) commonly used nowadays in TRM systems are costly and are produced using energy-intensive processes, and their end-of-life disposal entails a highly detrimental environmental impact. In addition, these types of textiles may not be suitable for the strengthening of relatively weak substrates such as historic masonry. This is because their high stiffness can cause premature cracking and/or slippage, precluding full exploitation of fiber strength. Natural fibers on the other hand are eco-friendly, renewable and have good economic feasibility. They also offer a higher degree of flexibility that precludes over-stiffening effects. In light of the above, researchers have been motivated to direct their efforts towards natural fiber inorganic matrix composites [8,9].

Despite the significant environmental and economic incentives that promote the development of natural TRM, there are several open issues in the engineering design of such systems. The mechanical behavior of textiles composed of natural fiber rovings differs significantly from that of textiles composed of man-made fibers. This is mainly due to the inferior strength and stiffness of natural fibers, the influence of crimping effects and the high variability in fiber properties. Furthermore, natural fibers show a high affinity to water and limited durability when exposed to alkaline environments. Although the design of brittle matrix composites has been studied in depth in the past and robust engineering analysis tools have been established, their applicability in the case of natural TRM has not yet been adequately explored. Instead, research is, at present, largely based on laboratory experimentation and empirical methods.

This paper aims to provide a framework for the engineering design of natural TRM composites with pseudo-strain-hardening and multiple cracking behavior. In order to assist in obtaining a more in-depth understanding of the mechanics of natural TRM, data from the literature regarding the characteristics of natural fibers is provided, while the synthesis and performance of composites examined in various studies are reviewed. A comparative analysis is performed between the properties of the constituents used in natural- and synthetic-fiber TRMs, with reference to both laboratory-tested and commercial systems. This enables the expression of practical guidelines concerning the selection of suitable matrix materials for natural TRMs. Finally, mechanics theories behind the design of continuous aligned fiber composites are presented and a rational approach for the theoretical design of natural TRM composites is proposed. The findings of this study enable identifying the specific problems that must be solved for advancing to real-life applications of natural TRMs in engineering. In this respect, paths for future research are also discussed.

## 2. Natural Fiber Reinforcement

**Figure 1:**
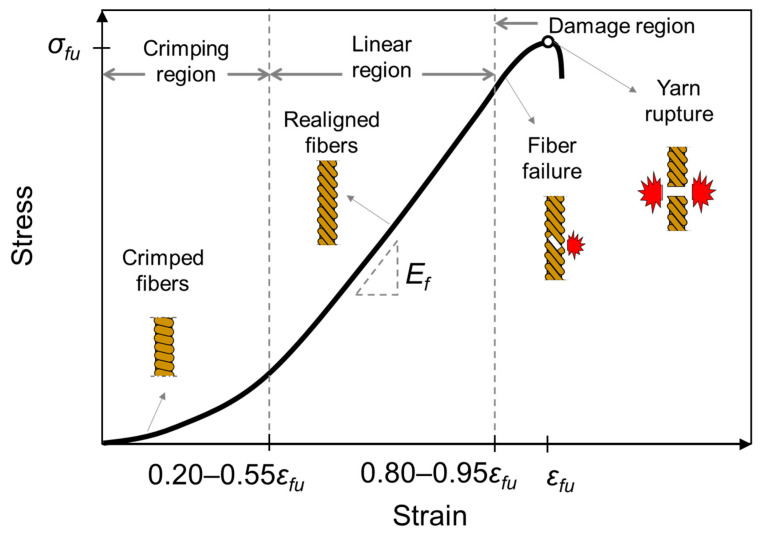
Typical stress–strain behavior of untreated natural fiber yarns under tension.

Reinforcement in natural TRM composites comprises yarns, which are usually formed by twisted bundles of fibers. Table 1 presents the mechanical properties of different types of vegetal fiber yarns obtained from the literature. The typical tensile behavior of untreated yarns is illustrated in Figure 1. The initial response is nonlinear and is characterized by low stiffness, which progressively increases. This phenomenon is associated with the straightening and realignment of the crimped twisted fibers along the loading axis [10]. After the realignment of the fibers, a linear region occurs in the stress–strain curve. This is followed by random damage of the fibers within the yarn until the tensile capacity is reached and the yarn ruptures.

The inherent inhomogeneity and variable crimping status of twisted natural fibers result in significant dispersion in the mechanical properties of untreated yarns (see Table 1) and introduce uncertainties in the definition of TRM design parameters [23,24]. Very often, the crimping effect is ignored, and the yarns are regarded as linear elastic materials with elastic moduli equal to the slope of the linear part of the stress–strain curve. However, experimental results [11] show that untreated yarns may have to be stretched to >20% of their deformation capacity before the fibers are adequately strained so that linear behavior is attained. The strain at which crimping effects terminate may thus exceed by an order of magnitude the tensile cracking strain of common inorganic mortars. From an engineering design point of view, this means that upon cracking of the TRM matrix, the actual effective modulus of a reinforcing yarn would be much lower than the nominal value assumed.

In terms of physical properties, untreated yarns exhibit a high affinity to water due to the hydrophilicity of their constituent fibers. Cellulose-rich vegetal fibers tend to absorb water rapidly and sustain considerable dimensional changes upon variation in the moisture content [25,26]. This can adversely affect the fiber–matrix interface properties [27] and, hence, the end-performance of the TRM composite. Furthermore, natural fibers are prone to degradation inside alkaline cement- and lime-based mortars [28,29,30,31]. In such environments, degradation mechanisms associated with the mineralization of the fibers and deterioration of the lignin and hemicellulose occur, causing a reduction in the fibers’ strength [11] and, subsequently, a decrease in the composite’s toughness and cracking density [32].

To address the aforementioned drawbacks, several fiber treatments have been considered. These include a wide range of treatments for imparting hydrophobicity [33,34], washing protocols for cleansing the fibers’ extractive content [16], and thermal and alkali treatments for partially reducing non-cellulosic components such as hemicellulose, lignin, wax and impurities [35,36,37]. The removal of non-cellulosic impurities is believed to be the primary mechanism of strength enhancement in treated natural fibers [38,39,40]. After the removal of these materials, the interfibrillar region can become less dense and less rigid, allowing the fibrils to rearrange more easily along the direction of tensile deformation. Alkali treatments have also been found to improve chemical and mechanical anchoring to cementitious matrixes by exposing more reactive groups of cellulose to bond with the mortar paste and by increasing the fibers’ surface roughness [40,41].

For increasing the initial stiffness and imparting a quasi-linear stress–strain response, the impregnation of yarns with polymers and resins has been considered [15,19,22]. Such coatings can penetrate the yarn structure, and as they harden and become more rigid, they create a surface layer with enhanced interlocking between the fibers thus producing a stiffening effect. Impregnated yarns behave as composites themselves, and their mechanical behavior is strongly influenced by the thickness of the coating layer [19]. In fact, relevant research has extended to the development of natural fiber-reinforced polymer bars that comprise multiple vegetal yarns impregnated in resin and are suitable for construction applications [42]. It should be noted that if the yarns are not adequately prestressed during the coating process, the twist and crimp of the fibers will remain upon impregnation and some reduction in the strength and deformation capacity will occur [43]. Furthermore, concerns are raised regarding the environmental impact of resin-based coatings. Recently, the use of tailored graphene/polyurethane nanocomposite coatings has been proposed [18]. This solution is promising as it can enhance the tensile properties and environmental resistance of yarns without compromising sustainability.

The natural textiles used in TRM fabrication typically have a woven structure with yarns arranged in the warp and weft directions forming a two-directional cross pattern (0°/90°). The tensile stress–strain behavior of untreated textiles is similar to that of individual yarns. Initially, the response is characterized by a nonlinear low stiffness zone due to decrimping and crimp interchange, while after the textile has been adequately stretched, the stiffness increases and the response becomes almost linear up to failure [44]. Composite element properties are influenced by the orientation and shape of the yarns within the textile [45]. Achieving higher reinforcing efficiency by controlling the alignment of the yarns so that these are straight and oriented in parallel with the loading direction can be challenging in natural TRMs. Untreated textiles have to be stretched for the yarns to remain straight during the fabrication process. Impregnation treatments can also result in dimensionally stable textiles. Certain coatings (e.g., resin-based impregnation), however, can cause over-stiffening and embrittlement, leading to damage to the textile when bent to fit on uneven surfaces. Furthermore, the impregnation of non-stretched yarns can lead to an irregular textile architecture. Stress development in the case of woven textiles is additionally affected by the yarns’ crimping angle [46]. The influence is more pronounced in textiles with a high yarn density [47] where the closely bunched yarns exhibit large angles with respect to the loading direction. Finally, yarn spacing affects the constructability of TRMs since it controls the penetrability of the textile by the matrix.

## 3. Synthesis and Performance of Natural TRM Composites

For the fabrication of TRM composites, natural fibers are commonly embedded in cement- or lime-based mortars. Many studies [13,14,15,22,48,49,50] have considered the use of premixed commercial mortars that may contain, in addition to the primary binder and aggregate, different additives (e.g., pozzolanas, geopolymers) and admixtures (e.g., workability aids, synthetic resins). Mortars with fine granulometry (max. grain size < 2 mm) are generally preferred to ensure adequate penetrability within the textile grid. Much work has focused on natural TRMs comprising a hydraulic lime matrix. This is because the intended application is often the strengthening of heritage masonry structures, and this type of mortar is considered to be compatible with such substrates [51]. Laboratory-developed cementitious mortars have also been used [52,53,54]. Certain researchers examined the addition of short discrete fibers in lime matrices, aiming to improve the post-crack behavior of TRM [55], while others considered the partial replacement of cement with pozzolanas (e.g., metakaolin, nanoclay) to reduce alkalinity and limit the deterioration of natural fibers [56,57]. The results show that dispersed fibers produce a bridging effect, which can lead to composites with denser crack patterns. The beneficial effects of dispersed fibers have been also verified by studies on hybrid cementitious composites [58,59,60]. However, their incorporation in the mortar matrix tends to decrease workability in the fresh state, making the use of plasticizing admixtures necessary. Pozzolanic additions in cementitious mortars were proven to reduce the calcium hydroxide phase, which is responsible for the degradation of the fibers’ cellulose. Nevertheless, in the case of untreated textiles, the degradation of other fiber components (primarily hemicellulose) cannot be prevented with this solution [56].

Pull-out tests [19] on flax yarns embedded in lime mortar show that debonding behavior is characterized by a linear response up to the maximum bond stress (adhesive bond phase), followed by a drop in the load resistance and a frictional phase with quasi-constant residual strength. In cases where some degree of mechanical bond exists (e.g., due to surface irregularities of the yarns), the peak load is preceded by a hardening branch. Table 2 gives the maximum bond stress values reported in the literature for natural fibers embedded in inorganic matrices. The results exhibit noticeable dispersion, being influenced by the fiber embedment length [61] and the high variability of the fibers’ geometric properties, which affects the mechanical/frictional bond [62]. Higher pull-out loads have been recorded after hornification, alkaline, peeling and nano-silica treatments [40,53]. Data regarding the effect that different coatings pose on bond behavior are somehow contradictory. Certain researchers [19,63] report a reduction in bond strength after coating with XSBR (carboxylate styrene butadiene rubber) and bio-based (cellulose acetate, cassava starch, hydrophobic starch) polymers. They have mainly attributed this behavior to the creation of a weak polymeric layer between the yarns/fibers and the mortar and to the lower surface roughness of coated yarns/fibers. Other researchers who considered XSBR and resin-based coatings [40,64,65] observed an improved response. Their results show that the interaction between XSBR and cementitious materials due to Ca^2+^ ions can enhance chemical and physical bonding [65], leading in some cases to a polymer-matrix bond that is even stronger than the fiber-coating bond [64]. Opposing trends may presumably be due to the particular characteristics of the fiber, matrix and coating materials used in various studies and to differences in the coating processes applied. The results may have been further influenced by the testing parameters adopted, as variations occur in the specimen types (cylindrical, prismatic, plated), clamping mechanisms, loading rates and embedment lengths considered. It should be underlined that the actual anchoring mechanisms of woven textiles are more complex than those of individual yarns/fibers. This is because the former is affected by the crimped geometry of the yarns within the textile, the frictional resistance at the warp–weft interlaces and the possible restraining action imposed by the yarns perpendicular to the load direction [66].

Table 3 presents the tensile properties of natural TRM composites, along with the characteristics of their constituent materials. Despite their variability, the results demonstrate that higher mechanical properties can be achieved by increasing the volumetric ratio of textile reinforcement and/or by using fibers with superior properties. Interestingly, the fiber reinforcement ratio in existing studies is not treated as an aspect of engineering design but rather as an experimental variable that is empirically modified until satisfactory results are obtained.

**Figure 2:**
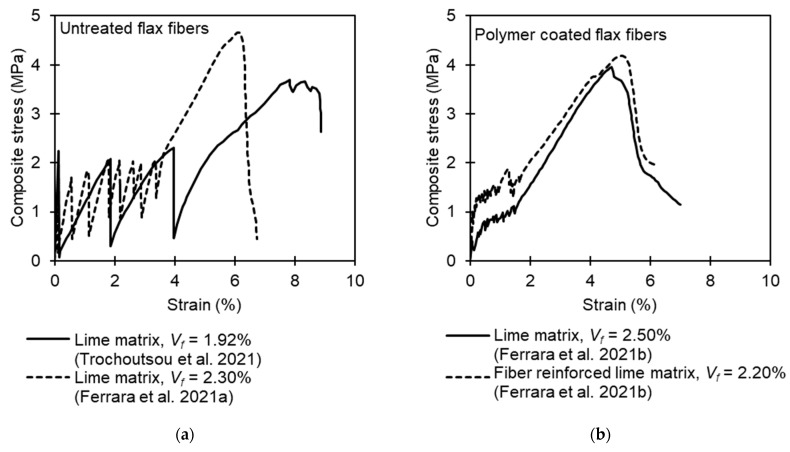
Tensile stress–strain diagrams adapted by Trochoutsou et al. [13] and Ferrara et al. [48,55]: (**a**) untreated flax textiles embedded in hydraulic lime mortar; (**b**) polymer-coated flax textiles embedded in hydraulic lime mortar with and without dispersed short fibers.

Figure 2 compares stress–strain curves obtained from tensile tests on TRM composites fabricated with untreated and treated flax textiles. The initial response is elastic up to the development of the first crack in the matrix. Given that an adequate volume of fiber reinforcement is provided, the elastic stage is followed by a multiple cracking zone in which a decrease in the composite’s stiffness is observed. After the complete development of the crack pattern, an almost linear hardening branch occurs up to failure. The response in the last stage is mainly governed by the properties of the textile and of the textile–matrix bond. Failure is usually either by rupturing of the yarns or by slippage of the textile within the matrix (telescopic failure). In composites with untreated fibers, a dramatic reduction in stress capacity (>70%) is observed upon the formation of the first crack. Even though specimens can eventually exhibit multi-cracking and pseudo-strain-hardening, experimental data indicate that upon crack initiation the load resistance may fall below an acceptable service limit. This phenomenon is probably associated with the particularly low initial elastic modulus of untreated fibers and with poor bonding between the untreated fibers and the matrix. The use of polymer-coated yarns was found to limit the load drops occurring after matrix cracking. Further improvement was attained by adding discrete short fibers in the matrix to impart post-crack tensile resistance.

A detailed review of the performance of natural TRM composites in strengthening applications can be found in Abbass et al. [69]. Promising results have been obtained in several studies considering natural TRM overlays on brick, stone and adobe masonry [17,70,71,72,73,74,75,76]. Overall, the experimental data show that such systems can potentially increase the bearing capacity and ductility of masonry elements under in-plane shear and eccentric axial loading and may also prevent brittle failure. The results obtained in certain studies [72,77], however, imply that the effectiveness of TRMs comprising untreated textiles can be hindered by slippage and/or delayed activation of the fiber reinforcement. The bonding of natural TRMs on masonry substrates has been found to be greatly influenced by the textile architecture and configuration [78]. The use of textiles with high yarn density and/or multiple textile plies can lead to poor matrix impregnation, thus promoting premature slippage and delamination phenomena.

## 4. Comparison between Natural TRM and Conventional Composite Systems

Inorganic matrix composites comprising synthetic (carbon, glass, polyparaphenylene benzobisoxazole (PBO)), mineral (basalt) and metallic (steel) reinforcement have been studied extensively in recent decades, both in the context of externally bonded strengthening applications and of thin element fabrication. Good practices regarding the composition of such systems have been established, and several commercialized systems have been developed. Given the background that exists on conventional composites, a comparative analysis is hereby presented aiming to define a framework concerning the characteristics that the constituents of natural TRM should meet. Table 4 summarizes data for basalt, carbon, glass, PBO and steel composites collected from twenty-five research studies [79,80,81,82,83,84,85,86,87,88,89,90,91,92,93,94,95,96,97,98,99,100,101,102,103] and from the specifications issued by twelve different material manufacturers (BASF, Biemme, CVR, Fassa Bartolo, FibreNet, G&P Intech, Kerakoll, Kimia, Mapei, Olympus, Ruregold, S&P, Sika) for 76 commercial systems. For each textile–matrix combination, the adopted reinforcing ratios (*V_f_*) and the resulting tensile strength (expressed as a function of the reinforcement area *σ_cu_/V_f_*) and failure strain (*ε_cu_*) of the composites are reported.

The mechanical properties of the matrix materials used in conventional systems typically satisfy certain performance criteria. Their compressive and flexural strengths are *f_m,c_* > 10 MPa and *f_m,fl_* > 2 MPa, respectively, to ensure adequate resistance against service loads. Their elastic modulus is generally an order of magnitude lower than that of the reinforcement to enable effective stress transfer to the fibers. The matrix type and composition mainly depend on the intended application. Lime-based matrices with *f_m,c_* = 10–15 MPa and *f_m,fl_* = 3–5 MPa are usually selected for systems to be applied on weak masonry substrates. Cement or geopolymer mortars with *f_m,c_* = 25–60 MPa and *f_m,fl_* > 3.5 MPa are often preferred for strengthening concrete structures. High-strength (*f_m,c_* > 60 MPa) cementitious mixtures are mostly used for the fabrication of thin structural/architectural components.

**Figure 3:**
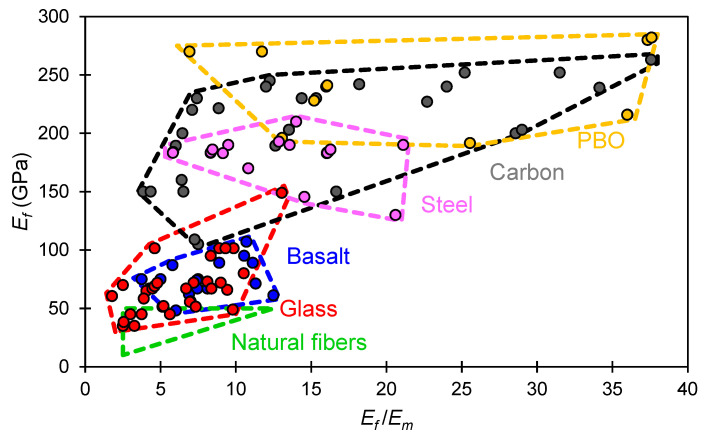
Elastic moduli of different reinforcement materials (*E_f_*) used in continuous fiber composite systems and respective ratios between the elastic moduli of composites’ reinforcement and matrix (*E_f_*/*E_m_*).

Figure 3 compares the ratios between the elastic moduli of the reinforcement and matrices for various conventional composite systems. The range *E_f_*/*E_m_* reported for natural fibers is theoretical and was obtained assuming that *E_f_*/*E_m_* > 2 has to be adopted and that *E_f_*/*E_m_* cannot exceed 12.5 since natural fibers exhibit a maximum *E_f_* in the range of 50 GPa, while a reasonable minimum value for *E_m_* is 4 GPa. Data show that the ratio *E_f_*/*E_m_*, which affects composite response at the pre-crack and crack formation stages, can vary between 2 and 38 for conventional systems. In most cases, ratios *E_f_*/*E_m_* = 5–15 are found.

Although synthetic, mineral and metallic fibers exhibit superior mechanical properties than vegetal ones, their high tensile capacity (>500 MPa) cannot always be exploited (see, for example, data for basalt and carbon textiles in Table 4). This is particularly true in strengthening applications where fiber strength utilization is influenced by the textile-to-matrix and matrix-to-substrate bond. Tests conducted on TRM composites applied on brick masonry substrates [89,104] have shown that the bond strength at the interfaces quite often is far lower than the tensile capacity of high-strength fibers (i.e., steel and PBO). This gives rise to debonding phenomena associated with cohesive failure of the substrate or failure along the matrix-to-substrate or textile-to-matrix interfaces. A comparative study [75] examining the performance of PBO and flax TRM systems also concluded that the use of high-strength synthetic fibers on weak masonry substrates is inadvisable as debonding is likely to precede textile failure, thus precluding full exploitation of the reinforcement’s tensile capacity. A more ductile bond-slip response with a higher fiber strength exploitation ratio was observed for flax TRM systems, indicating that these are more compatible with masonry. Nevertheless, the relatively lower strength of natural textiles can be a limiting factor in concrete strengthening applications. Flexural tests performed on concrete beams showed that synthetic textiles can significantly increase the members’ post-yield moment capacity, despite the fact that the strengthening action can still be influenced by debonding and slippage phenomena [105,106]. Analogous experiments conducted using hemp TRM systems revealed that the tensile capacity of natural textiles can be reached at load levels near the member’s yielding point, thus restricting strengthening action to the ultimate service limit state [107]. In terms of stiffness, natural textiles are only comparable with certain types of glass and basalt textiles that have elastic moduli of 35–60 GPa. All other textile reinforcements used in conventional TRM systems have systematically higher elastic moduli.

Based on the above, it can be argued that matrices used in natural TRM should be tailored to give a ratio *E_f_*/*E_m_* > 2, while satisfying a threshold-bearing capacity *f_m,c_* > 6 MPa. Matrix materials with elastic moduli in the range 4–10 GPa are likely to be suitable for textiles composed of coated flax and hemp yarns with elastic moduli of 10–50 GPa. This points towards the use of lime-based or low-strength cement mortars. Meeting the aforementioned criteria in the case of low-stiffness natural textiles can be difficult in practice. This is because modifying the mortar’s mix design to substantially reduce the elastic modulus will most probably have an adverse effect on the material’s bearing capacity. In this respect, achieving an acceptable *E_f_*/*E_m_* ratio without compromising strength is possibly unrealistic for jute, sisal, coir and cotton textiles with elastic moduli < 10 GPa.

## 5. Engineering Design of Natural TRM Composites

In the following, some basic considerations for the design of natural TRM composites with strain hardening and multi-cracking behavior are discussed. The analytical models hereafter proposed are mostly based on the well-established theories formulated by Aveston, Cooper and Kelly [108]. These researchers have used rule-of-mixtures and fracture mechanics concepts to describe the behavior of composite materials. The following basic assumptions have been made:

- The tensile response of both the matrix and the fibers is linear elastic–perfectly brittle and is characterized by (deterministic) fixed values of elastic modulus (*E_m_* and *E_f_*), tensile strength (*σ_mu_* and *σ_fu_*) and failure strain (*ε_mu_* and *ε_fu_*).
- The fibers are aligned parallel to one another and are uniformly distributed throughout the matrix.
- Forces are applied parallel to the fiber direction (i.e., the fiber reinforcement carries load only along the loading axis).
- The matrix is free of voids and the quantity of the fiber reinforcement (*V_f_*) does not pose any influence on the porosity of the matrix, so the volumetric proportions of the two constituents are related as *V_m_* = 1 − *V_f_*.
- The composite is initially in a stress-free state, and there are no residual stresses arising from shrinkage phenomena, thermal expansion–contraction, etc.
- Poisson effects can be neglected.
- In the pre-crack state, there is perfect bonding between the fibers and the matrix, and hence, the strains on the fiber and matrix are equal, while the stresses are proportional to each constituent’s elastic modulus.
- After a crack develops in the matrix and reaches the fibers, debonding at the fiber–matrix interface will occur.
- After debonding, stress transfer at the fiber–matrix interface is governed by friction only with a constant frictional bond shear strength (*τ_s_*). It should be underlined that *τ_s_* has no physical significance; it is merely a fictitious averaged bond shear strength that enables treating the complex stress-transfer problem in the context of practice-oriented design. It is also emphasized that the value of *τ_s_* should not the confused with the maximum bond stress reported in Table 2.

Further details regarding the assumptions made when using the rule-of-mixtures for the calculation of the mechanical properties of composites prior to crack formation can be found in [109,110].

### 5.1. Critical Fiber Volume to Control Stress Transfer upon Cracking

In natural TRM composites where the failure strain of the matrix is typically much lower than the failure strain of the fibers (*ε_mu_* << *ε_fu_*), the first crack occurs when the tensile capacity of the matrix (*σ_mu_*) is reached. Based on the assumption of elastic stress distribution up to crack formation, the composite stress at the first crack (*σ_c_*_1_) is given by [111]:

*σ*_*c*1_ = *σ_mu_ V_m_* + *σ*’*_f_ V_f_* (1)

where *σ’_f_* is the tensile stress of the fibers, which can be estimated as *σ’_f_* = *E_f_ ε_mu_* = *σ_mu_* (*E_f_*/*E_m_*) from strain compatibility.

After cracking of the matrix, the load will be thrown onto the fibers. If failure is to be prevented at this stage, the load-carrying capacity of the fiber reinforcement must be greater than the load on the composite at first crack [112], i.e., *σ_fu_ V_f_ ≥ σ_c_*_1_. The critical fiber volume can thus be calculated as:

(2) $$V_{f,\mathrm{crit}}\geq \frac{\sigma_{\mathrm{mu}}}{\sigma_{\mathrm{fu}}+\sigma_{\mathrm{mu}}\left(1-\frac{E_{f}}{E_{m}}\right)}$$

### 5.2. Critical Fiber Volume to Control Crack Spacing

**Figure 4:**
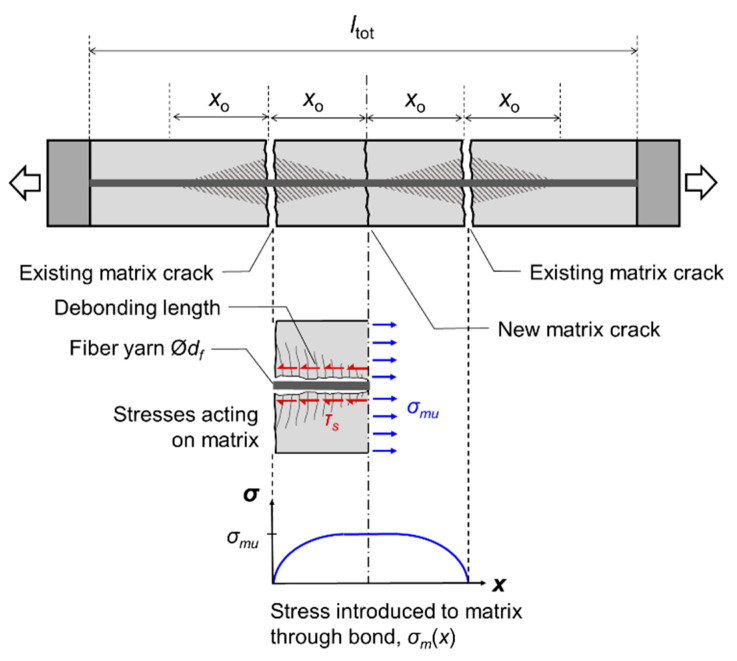
Distribution of stresses acting on the matrix between two cracks.

The volumetric ratio of fiber reinforcement should be adequate to ensure that the crack spacing achieved is short enough for multiple cracks to develop along the specimen’s length. The debonding length *x_o_*, which is the distance from a crack face at which the matrix stress reaches its tensile capacity, is obtained from equilibrium (Figure 4):

(3) $$x_{o}=\frac{\sigma_{\mathrm{mu}}d_{f}V_{m}}{4{\tau_{s}V}_{f}}$$

For *i* ≥ 2 number of cracks to develop in a composite specimen (*i* = 2 cracks being the minimum condition for multi-cracking), the sum of the debonding lengths on either side of each crack should be lower than the total specimen length, i.e., 2*ix_o_* ≥ *l_tot_*. Based on the above, the critical fiber volume to control crack spacing is:

(4) $$V_{f,\mathrm{crit}}\geq \frac{i\sigma_{\mathrm{mu}}d_{f}}{{i\sigma_{\mathrm{mu}}d}_{f}+2l_{\mathrm{tot}}\tau_{s}}$$

Evidently, Equation (4) has some practical significance in the design of tensile specimens of limited length (*l_tot_* < 500 mm). In actual TRM applications where the lengths of composite layers are sufficiently large (typically in the order of meters), adequate frictional resistance can theoretically develop along the fiber–matrix interface to enable the formation of multiple cracks and to allow mobilization of the fiber strength.

### 5.3. Critical Fiber Volume to Limit the Drop in Load Resistance upon Formation of the First Crack

Under deformation-controlled tensile loading, the formation of the first crack is accompanied by a drop in load resistance. As the fibers bridge the crack, the load resistance increases again up to the formation of the next crack. Then, the load drops and increases again, and this is repeated until a crack saturation state is reached. Notably, the load drops observed in some studies examining natural TRM composites [13,48,49] are as high as 80% (see Figure 2). Therefore, the fiber volume provided should ensure sufficient residual strength after the formation of the first crack.

**Figure 5:**
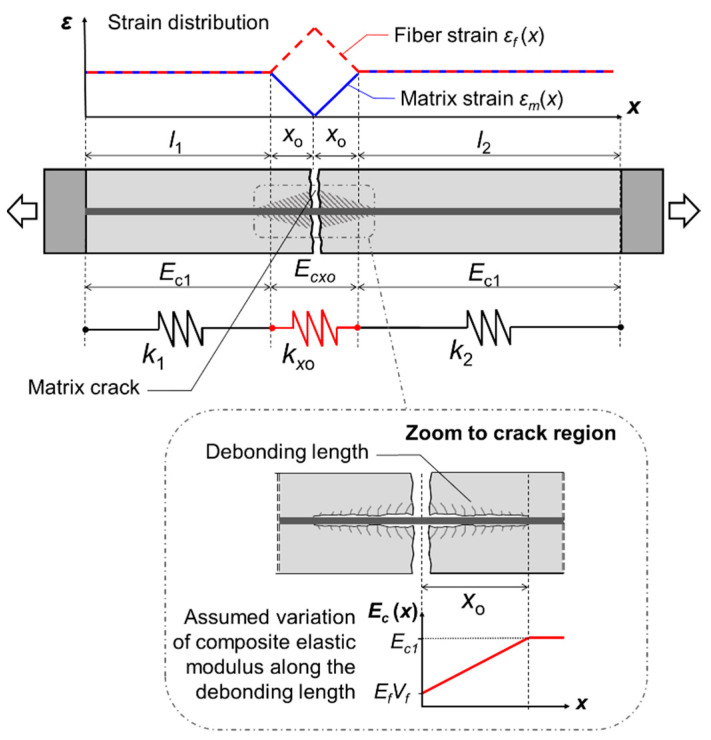
Schematic representation of the model used for determining the equivalent stiffness of the composite after the formation of the first matrix crack. The cracked stiffness is used for calculating the drop in load resistance upon cracking.

The method hereby proposed for estimating the fiber volume required to limit the drop in load resistance upon formation of the first crack is based on the model by Saidi and Gabor [95]. The latter assumes that the drop in load resistance depends on the reduction in the stiffness of the cracked specimen (i.e., on the global elastic modulus of the cracked composite). The cracked stiffness is a function of the specimen length, the volumetric ratios and elastic moduli of the matrix and fibers, and the debonding length. A schematic representation of the adopted model is given in Figure 5.

In the pre-cracking zone, the response of the composite is assumed to be elastic, and the initial elastic modulus (*E_c_*_1_) is obtained from the rule of mixtures:

(5) $$E_{c1}=E_{m}V_{m}+E_{f}V_{f}$$

After the formation of the first crack, the composite comprises three parts:

- A section that extends on both sides of the crack by a load transfer length *x_o_*, over which debonding occurs. The local elastic modulus along the debonded interface (*E_cxo_*) will be lower than the initial elastic modulus of the composite (*E_c_*_1_). The part with a total length of 2*x_o_* is characterized by a stiffness of *k_xo_*.
- Two sections over which the fibers are fully bonded to the matrix and strain compatibility remains. The local elastic modulus along these two sections is equal to the initial elastic modulus of the composite (*E_c_*_1_). The two sections have lengths *l*_1_ and *l*_2_ and their equivalent stiffnesses are *k*_1_ and *k*_2_.

The three parts can be considered as springs connected in a series. For a specimen with cross-sectional area *A_c_* and total length *l_tot_* = *l*_1_ + *l*_2_ + 2*x_o_*, the global equivalent stiffness after the formation of the first crack (*k_eq_*) is given by:

(6) $$\frac{1}{k_{\mathrm{eq}}}=\frac{l_{\mathrm{tot}}}{A_{c}E_{c2}}=\frac{1}{k_{1}}+\frac{1}{k_{2}}+\frac{1}{k_{\mathrm{xo}}}=\frac{l_{1}}{A_{c}E_{c1}}+\frac{l_{2}}{A_{c}E_{c1}}+\frac{2x_{o}}{A_{c}E_{\mathrm{cxo}}}$$

In the above equation, *E_c_*_2_ is the global elastic modulus of the composite after the formation of the first crack.

For estimating the local elastic modulus of the composite over the length *x_o_*, it is considered that the matrix does not contribute to the stiffness at the crack plane. At this position, the elastic modulus reduces to the equivalent modulus of the fiber reinforcement alone (*E_f_V_f_*). Moving away from the crack position, the matrix strain and the contribution of the matrix to the stiffness are assumed to increase and the elastic modulus reaches the nominal value *E_c_*_1_ at *x_o_*. The evolution of the composite’s elastic modulus along the debonding length (0 ≤ *x* ≤ *x_o_*) is assumed to be linear and can be described by the equation:

(7) $$E_{c}\left(x\right)=\frac{E_{m}V_{m}}{x_{o}}x+E_{f}V_{f}$$

The equivalent stiffness along the debonding length can be calculated considering this section as an assembly of infinitesimal elements of length *dx* connected in a series:

(8) $$\frac{1}{k_{\mathrm{xo}}}=\frac{x_{o}}{{A_{c}E}_{\mathrm{cxo}}}=\int_{0}^{x_{o}}\frac{\mathrm{dx}}{A_{c}E_{c}\left(x\right)}$$

Using Equations (7) and (8), the following expression is derived for the local elastic modulus over the debonding length:

(9) $$E_{\mathrm{cxo}}=\frac{E_{m}V_{m}}{\ln\left(\frac{E_{c1}}{E_{f}V_{f}}\right)}$$

The global elastic modulus of the composite specimen after the formation of the first crack (*E_c_*_2_) is obtained by:

(10) $$E_{c2}=\frac{l_{\mathrm{tot}}E_{c1}E_{\mathrm{cxo}}}{2x_{o}E_{c1}+E_{\mathrm{cxo}}(l_{\mathrm{tot}}-2x_{o})}$$

By assuming that crack propagation is instantaneous so that the global strain in the specimen just before and just after the formation of the first crack is the same, the stress drop (*σ_cdrop_*) upon cracking can be attributed to a reduction in the global elastic modulus:

(11) $$\varepsilon_{c1}=\frac{\sigma_{c1}}{E_{c1}}=\frac{\sigma_{c1}-\sigma_{\mathrm{cdrop}}}{E_{c2}}$$

From Equation (11) the reduction in the load resistance of the composite is estimated as:

(12) $$\frac{\sigma_{\mathrm{cdrop}}}{\sigma_{c1}}=1-\frac{E_{c2}}{E_{c1}}$$

The critical fiber volume for limiting the drop in load resistance upon cracking (*V_f,crit_*) is obtained using Equations (5), (9), (10) and (12) by setting an acceptable limit for the ratio *σ_cdrop_*/*σ_c_*_1_.

### 5.4. Control of Energy Absorption during Multiple Fracture

Using fracture mechanics concepts, it can be shown that fiber reinforcement can cause crack suppression in the matrix. In principle, cracking under tensile loading will occur when the failure strain of the matrix (*ε_mu_*) is reached and provided also that there is a decrease in the potential energy of the composite specimen and the loading system. Considering the energy changes taking place when a crack is formed under conditions of fixed load (work performed by the applied stress, loss of strain energy in the matrix, increase in the fiber strain energy, work performed by frictional forces due to relative movement of the fibers in the matrix) and assuming a pure frictional fiber–matrix bond, Aveston, Cooper and Kelly [108] showed that if the fracture surface work in forming a crack in the matrix is *γ_m_*, then a crack in the composite will only form if:

(13) $$2\gamma_{m}V_{m}\leq \frac{E_{c}E_{f}\varepsilon_{\mathrm{mu}}^{3}{\left(\frac{E_{m}V_{m}}{E_{f}V_{f}}\right)}^{2}d_{f}}{12\tau_{s}}$$

From Equation (13), it can be deduced that there is an upper limit of fiber content above which cracking will not occur at the normal failure strain of the matrix *ε_mu_*, but the composite cracking strain will have to be increased to a strain *ε_muc_* that is given by:

(14) $$\varepsilon_{\mathrm{muc}}={\left(\frac{{24\tau_{s}\gamma_{m}E}_{f}V_{f}^{2}}{E_{c}E_{m}^{2}d_{f}V_{m}}\right)}^{1/3}$$

Crack suppression in the matrix and multiple fracture will occur as long as the increased cracking stress (*σ’_c_*_1_) does not exceed the capacity of the fiber reinforcement:

*σ’_c_*_1_ = *E_c_ε_muc_* < *σ_fu_ V_f_* (15)

In cases where the selected fiber content is high enough to result in increased cracking strain, the validity of Equation (15) should be verified as this marks the transition from multiple to single fracture.

### 5.5. Prediction of Tensile Stress–Strain Response

**Figure 6:**
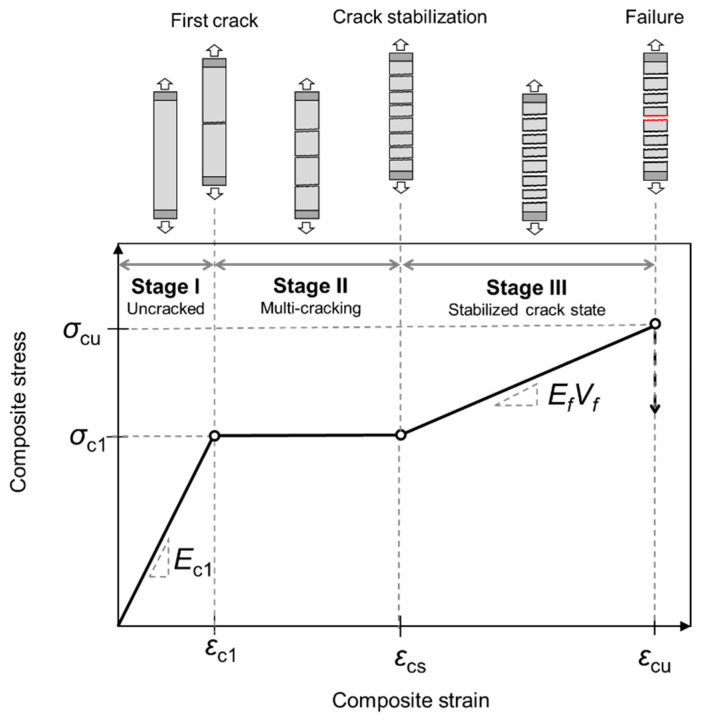
Trilinear representation of the tensile stress–strain response of a strain-hardening unidirectional composite according to the ACK model.

Figure 6 shows the Aveston–Cooper–Kelly (ACK) model describing the tensile stress–strain behavior of strain-hardening unidirectional composites. The model gives a simplified trilinear representation of the response under tension. The following three stages are identified:

- Stage I: The matrix is uncracked and a perfect bond between matrix and fabric is assumed. The elastic modulus of the uncracked composite is obtained by the rule of mixtures as per Equation (5). Stage I ends when the first crack occurs. The composite stress at the first crack can be calculated from Equation (1) or Equation (15), depending on whether the amount of fiber reinforcement can cause crack suppression in the matrix or not.
- Stage II: Provided that the fiber content is adequately high (see Section 5.1), the matrix material will exhibit multiple cracking until it reaches a crack stabilization (i.e., crack saturation) state. The ACK model assumes that the multiple cracking process continues at constant composite stress. Assuming that, in the post-crack state, a constant frictional shear stress develops along the debonded length *x_o_*, the crack stabilization state is reached at a composite strain:
  (16) $$\varepsilon_{\mathrm{cs}}=\left(1+a\frac{E_{m}V_{m}}{E_{f}V_{f}}\right)\varepsilon_{\mathrm{mu}}$$
  Parameter *a* takes values between ½ and ¾ and is commonly taken as *a* = 0.666 based on a theoretical average crack saturation spacing of 1.337*x_o_* [113,114].
- Stage III: Additional loading after the crack stabilization stage will cause the fibers to stretch up to failure. Hence, the ultimate tensile strength of the composite (*σ_cu_*) can be taken as equal to the tensile capacity of the embedded fiber reinforcement:
  *σ_cu_ =σ_fu_ V_f_* (17)
  Assuming that the modulus of elasticity in the post-multiple cracking zone is *E_f_V_f_* (i.e., there is no contribution of the matrix to the global stiffness), the ultimate failure strain of the composite (*ε_cu_*) will depend on the ultimate strain of the fiber and the crack spacing:
  (18) $$\varepsilon_{\mathrm{cu}}=\varepsilon_{\mathrm{fu}}-\varepsilon_{\mathrm{mu}}\frac{E_{m}V_{m}}{E_{f}V_{f}}(1-a)$$

### 5.6. Design Example

The proposed methodology for calculating the required fiber reinforcement is hereby presented through a theoretical example. A hydraulic lime mortar with an elastic modulus of *E_m_* = 9.5 GPa and a flexural strength of *f_m,fl_* = 3.5 MPa is considered as the matrix material. The uniaxial tensile strength of the matrix is approximated from the flexural strength considering a typical mortar specimen with depth *h_b_* = 40 mm; *σ_mu_* = *f_m,fl_* [0.06*h_b_*^0.7^/(1 + 0.06*h_b_*^0.7^)] = 1.5 MPa [115]. The fracture energy of the matrix is taken as *G_m_* = 26.3 N/m, which gives a surface energy of *γ_m_* = *G_m_*/2 = 13.15 N/m. The fiber reinforcement consists of hemp yarns with a tensile strength of *σ_fu_* = 250 MPa and a nominal diameter of *d_f_* = 1 mm. Two cases are examined: the use of treated fibers with an elastic modulus of *E_f_* = 20 GPa, and the use of untreated fibers with an elastic modulus of *E_f_* = 4 GPa. A composite tensile specimen with a free length of *l_tot_* = 350 mm is assumed. The allowable drop in load resistance after the formation of the first crack is taken as *σ_cdrop_*/*σ_c_*_1_ = 20%.

**Figure 7:**
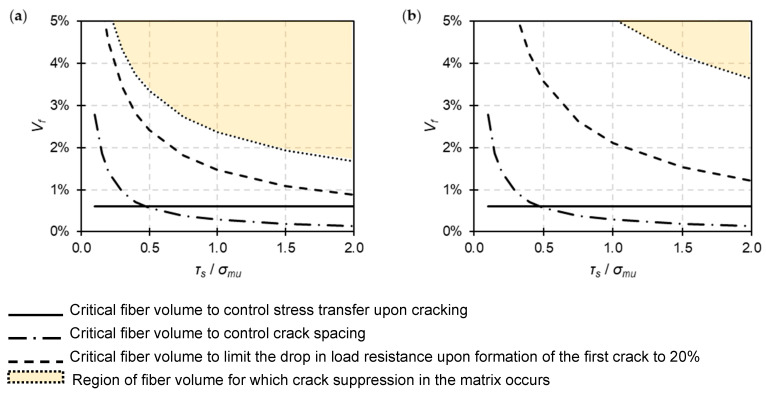
Fiber volumes required to control stress transfer upon cracking, crack spacing, residual resistance upon crack formation and crack suppression in the matrix as a function of the ratio between the fiber–matrix frictional bond shear strength and the matrix tensile strength. (**a**) Treated fibers with *E_f_* = 20 GPa and (**b**) untreated fibers with *E_f_* = 4 GPa.

Figure 7 presents the minimum volumetric proportions of fiber reinforcement required to achieve strain-hardening behavior with controlled crack spacing and adequate residual strength upon crack initiation. It also shows the quantity of fiber reinforcement above which crack suppression in the matrix will occur. The results are reported as a function of the fiber–matrix bond shear strength *τ_s_*. The latter is assumed to take values from 0.1 up to 2 times *σ_mu_*. It is indicatively noted that Eurocode 2 [116] recommends ratios *τ_s_*/*σ_mu_* in the range 0.9 to 1.9 to describe the shear resistance of the interface between concrete and steel reinforcement (the lower ratio refers to bars with an effectively plain surface and the higher ratio refers to high-bond ribbed bars).

According to the analysis, *V_f_* > 0.61% is, in both cases, adequate to prevent failure of the composite after the matrix cracks. For *τ_s_*/*σ_mu_* < 0.5, the volume of fiber reinforcement required to control crack spacing becomes higher than that required to avoid failure after cracking. Nevertheless, for the entire range of *τ_s_*/*σ_mu_* ratios considered, it was found that a substantially higher volume of fiber reinforcement is required to limit the drop in load resistance upon crack formation. This is particularly true for low-modulus untreated fibers, where achieving *σ_cdrop_*/*σ_c_*_1_ ≤ 20% entails a more than twofold increase in the critical fiber volume computed from Equation (2). In fact, for *τ_s_*/*σ_mu_* < 0.5, the volume of untreated fibers required to achieve adequate residual capacity upon cracking is *V_f_* > 3.5%. Therefore, it can be argued that poor shear bond strength is to a large degree responsible for the high drops in load resistance that were observed in certain studies (see Figure 2a), despite the use of rather high fiber volumes (*V_f_* ~ 2%). The calculations also show that crack suppression in the matrix is not likely to occur with the type of untreated fiber hereby considered, as the volumetric ratios of reinforcement required to impart such behavior are unrealistically high, even for a good fiber–matrix bond (*V_f_* > 3.6% for *τ_s_*/*σ_mu_* = 2).

The design example also introduces some interesting constructability aspects. When *τ_s_*/*σ_mu_* < 1.4, the demand in untreated fiber reinforcement becomes *V_f_* > 1.6%. This means that for the construction of a typical 10 mm-thick TRM overlay, a hemp textile with mesh spacing < 5 mm should be used. Such low mesh spacing is impractical as it will impede effective penetration from the mortar matrix. Hence, multiple plies of textile will have to be used. Using treated fibers, a single-ply TRM with *V_f_* < 1.6% can be realized when *τ_s_*/*σ_mu_* > 0.8.

Overall, the results highlight the influence that the fiber–matrix interface properties have on the response of the composite. Adequately high bond resistance is necessary to achieve multi-cracking behavior and to prevent the load resistance from dropping below an acceptable serviceability limit upon matrix cracking, while reasonably limiting the volumetric ratio of fiber reinforcement. It should be emphasized, however, that excessively high interfacial bond strength can lead to premature fiber rapture, precluding strain-hardening behavior. Further discussion on this issue can be found in Li and Wu [117], where a fracture mechanics approach is used to show that, for fixed fiber and matrix properties, there is an upper limit on the frictional bond strength.

## 6. Conclusions and Recommendations for Future Research

Natural fibers constitute a sustainable alternative to man-made fibers and have good potential for the fabrication of inorganic matrix composites used in construction. At this stage, however, the realization of natural TRM systems that can be safely adopted in real-life structural applications remains a challenging task, and further research is required. This paper highlighted critical issues that need to be addressed in this direction. Based on the analysis of the data presented, the following future research trends are identified:

- A framework for the rational engineering design of natural TRM composites should be formulated. This should become the theoretical basis of experimental campaigns dealing with natural composites. In this study, some of the classical mechanics and fracture mechanics theories for the analysis of continuous aligned fiber composites have been introduced. Such theories constitute a good starting point for the development of design tools tailored to natural TRM. More advanced computational models should also be explored. Design methods must account for the particular mechanical behavior of natural fibers and must be validated against experimental data.
- The development of suitable fiber treatments is essential for overcoming durability problems, for eliminating the initially nonlinear tensile response of crimped yarns and for imparting dimensional stability to textiles. In this respect, emphasis should be placed on coating protocols that can achieve the fiber mechanical response required for functional TRM systems. Particular reference is made to graphene/polyurethane nanocomposite coatings as these have better environmental merits than epoxy- and resin-based impregnation. Upscaling of coating methods from the laboratory to the industrial scale is also necessary for advancement to commercialized TRM systems.
- Matrix materials compatible with natural fibers should be developed. Mortars suited to natural TRM should have a low elastic modulus but should also possess an adequate bearing capacity to ensure good performance under service loads. Different additives and admixtures can be considered for improving the bond behavior and for reducing alkalinity to prevent fiber deterioration. Another research direction lies in the design of hybrid systems comprising textiles embedded in matrices reinforced with dispersed short fibers. The addition of discrete fibers in the TRM matrix is expected to enhance the composite’s load-deformation and energy absorption characteristics, resulting in improved post-crack behavior. This solution can promote the use of natural fibers in the form of dispersed mortar reinforcement and can also limit the significant load drops that tend to occur upon the cracking of natural TRM.
- The fiber–matrix bond behavior should be studied in depth since the interface properties influence the end performance of the composite to a great extent. To this end, the development of TRM systems should include the implementation of pullout tests at different embedment lengths. Relevant experimental data will assist in the derivation of useful design parameters (e.g., the critical bond length beyond which fiber failure prevails over debonding, the value of the frictional bond strength, etc.) and may also be utilized in the context of more rigorous analysis for the derivation of bond-slip laws.
- Alternative typologies of composites should be examined in order to overcome constructability issues arising in cases where high quantities of fiber reinforcement are required. A possible solution lies in the design of composite-reinforced mortar (CRM) systems. Compared to TRM systems that comprise textiles with close yarn spacing, CRM systems comprise preformed meshes composed of impregnated fiber bars arranged in a relatively wide pitch. The development of such reinforcing systems can benefit from research on the fabrication of natural yarn bars.

## Figures and Tables

**Table 1:** Tensile strength (*σ_fu_*), elastic modulus (*E_f_*) and tensile failure strain (*ε_fu_*) of dry natural fiber yarns considered in different studies as reinforcement in composites.

Fiber	Condition	*σ_fu_* (MPa)	*E_f_* (GPa)	*ε_fu_* (%)	References
Flax	Untreated	195–417	5.5–25	1.6–6.2	[11,12,13,14,15,16,17]
	Treated ^WT^	200–375	8–11	2.0–3.5	[16,18]
	Impregnated ^RC, PC, NC^	220–631	9–38	1.3–3.6	[15,18,19]
Hemp	Untreated	120–296	3–27	1.0–5.4	[14,15,16]
	Treated ^WT^	150–350	4–12	2.0–3.0	[16,18]
	Impregnated ^RC, NC^	460–545	21–51	0.95–2.30	[15,18]
Jute	Untreated	75–225	2.7–10	0.02–7.0	[13,14,20,21]
	Treated ^WT, AT^	36–102	0.5–1.5	5.0–12.0	[21]
	Impregnated ^PC^	88	4.5	2.3	[20]
Sisal	Untreated	96–323	1.8–8	0.05–4.9	[12,14,15,16,22]
	Treated ^WT^	220–320	5.5–9	3.0–4.0	[16]
	Impregnated ^RC^	92–350	3.7–9	2.1–2.5	[15,22]
Cotton	Untreated	53	0.5	10	[15]
	Impregnated ^RC^	82–92	0.8–0.9	7–8	[15]
Coir	Untreated	51	0.3	0.16	[14]

RC = resin coating; PC = polymer coating; NC = nanocomposite coating; WT = washing treatment; AT = alkali treatment.

**Table 2:** Maximum bond stress values from pull-out tests on yarns and single fibers embedded in inorganic matrices.

References	Matrix	Fiber			Embedment Length (mm)	Maximum Bond Stress (MPa)
		Type	Treatment	*d_f_* (mm)		
[19]	Hydraulic lime	Flax	Untreated	1.56	25	0.30–0.40
			Polymer coated	1.00	25	0.15–0.30
[64]	Hydraulic lime	Hemp	Untreated	1.30	10–40	1.0–2.2
			Polymer coated	1.48	10–40	1.6–3.3
			Resin coated	1.60	30	>2.0
[67]	Geopolymer	Hemp	Untreated	0.55	10–20	0.80
[63]	Cement	Sisal *	Untreated	0.20	20–40	0.15–0.19
			Biopolymer coated	0.23	20–40	0.10
[40]	Cement	Sisal *	Untreated	0.66	25	0.18
			Hornified	0.26	25	0.27
			Alkali treated	0.45	25	0.32
			Polymer coated	0.75	25	0.34
			Hornified + Polymer coated	0.85	25	0.31
[65]	Cement	Sisal *	Untreated	0.10	25	0.24
			Polymer coated	0.10	25	0.49
		Curauá *	Untreated	0.17	25	0.21
			Polymer coated	0.17	25	0.52
		Jute *	Untreated	0.07	25	0.39
			Polymer coated	0.07	25	0.78
[62,68]	Cement	Jute *	Untreated	0.27	5–10	0.25–0.40
			Hot water immersion	0.27	5–10	0.30–0.34

* Tests conducted on single fibers.

**Table 3:** Characteristics of matrix (compressive strength—*f_m,c_*, tensile strength—*σ_mu_*, elastic modulus—*E_m_*) and natural fiber reinforcement (textile area density—*ρ_s_*, tensile strength—*σ_fu_*, elastic modulus—*E_f_*) used in different studies and corresponding properties of the composite (volumetric ratio of fiber reinforcement—*V_f_*, initial elastic modulus—*E_c_*_1_, tensile strength—*σ_cu_*, exploitation ratio of the fiber reinforcement’s tensile strength—*σ_cu_*/*V_f_ σ_fu_*).

References	Matrix				Reinforcement				Composite Properties			
	Type	*f_m,c_* (Mpa)	*σ_mu_* (Mpa)	*E_m_* (Gpa)	Type	*ρ_s_* (g/m^2^)	*σ_fu_* (Mpa)	*E_f_* (Gpa)	*V_f_* (%)	*E_c_*_1_ (Gpa)	*σ_cu_* (Mpa)	*σ_cu_*/*V_f_ σ_fu_*
[13]	Lime	7.7	2.8 ^FL^	3.4	Flax	215	213	10.1	1.11	n/a	0.91	38%
									0.67	0.96	0.39	27%
									1.48	0.43	1.23	39%
									1.67	n/a	1.49	42%
					Flax	300	378	14.4	1.44	2.59	2.56	47%
									0.86	n/a	1.92	59%
									1.92	2.04	3.48	48%
									2.16	1.45	4.43	54%
					Jute	183	196	10.3	0.52	2.57	0.33	32%
									0.70	1.52	0.46	33%
									0.79	0.91	0.58	38%
[15]	Cement	39.3	4.6	8.9	Hemp ^RC^	750	521	38.7	1.26	0.28	6.98	100%
					Hemp ^RC^	990	544	50.6	1.26	0.16	5.99	87%
					Flax ^RC^	770	631	36.0	1.26	0.18	5.24	66%
					Flax ^RC^	1070	517	38.0	1.26	0.13	5.53	85%
					Sisal ^RC^	2170	111	4.9	7.85	1.33	7.38	85%
					Sisal ^RC^	2500	92	3.8	7.85	0.71	3.38	47%
					Cotton ^RC^	1890	92	0.93	5.65	0.34	5.42	100%
					Cotton ^RC^	2150	82	0.88	5.65	0.40	5.25	100%
[12,49]	Lime	14.6	1.0	n/a	Flax	375	397	9.9	1.32	0.30	2.22	42%
									1.35	3.65	2.65	49%
									2.11	0.32	3.84	46%
									2.70	3.81	8.32	78%
									4.05	4.29	12.88	80%
					Sisal	388	323	8.1	1.58	3.81	2.78	54%
									1.82	0.25	2.37	40%
									2.91	0.35	3.87	41%
									3.16	4.30	7.55	74%
									4.73	4.21	10.64	70%
[17,48,55]	Lime	9.5	4.2	n/a	Flax	300	331	12.5	1.30	n/a	1.56	36%
									2.30	n/a	4.51	59%
					Flax ^PC^	n/a	266	12.4	1.60	n/a	2.86	67%
									2.50	n/a	4.35	65%
	Lime ^DF^	9.9	2.9	n/a	Flax ^PC^	n/a	266	12.4	2.20	n/a	4.20	72%
	Lime	11.1	3.1 ^FL^	n/a	Flax	n/a	354	9.36	2.04	n/a	4.16	58%
									2.55	n/a	5.07	56%
[22]	Lime	13.0	3.5 ^FL^	n/a	Sisal ^AT^	Yarns	240	7.9	1.39	n/a	2.00	60%
[14]	Lime	12.2	3.1 ^FL^	n/a	Jute	255	102	4.5	n/a	n/a	n/a	25%
					Jute	1099	225	3.7	n/a	n/a	n/a	16%
					Hemp	454	164	4.6	n/a	n/a	n/a	20%
					Flax	388	198	5.9	n/a	n/a	n/a	29%

Superscripts: DF = contains discrete fibers; FL = flexural tensile strength; RC = resin coated; PC = polymer coated; AT = alkali treated. n/a = not available data

**Table 4:** Characteristics of textiles (tensile strength—*σ_fu_*, elastic modulus—*E_f_*) and matrices (compressive strength—*f_m,c_*, flexural strength—*f_m,fl_*, elastic modulus—*E_m_*) used in conventional TRM systems.

Textile			Matrix				Composite		
Type	*E_f_* (GPa)	*σ_fu_* (MPa)	Type(s)	*f_m,c_* (MPa)	*f_m,fl_* (MPa)	*E_m_* (GPa)	*V_f_* (%)	*σ_cu_*/*V_f_* (MPa)	*ε_cu_* (%)
Basalt	48–183	870–3080	Lime	11–21	3.2–6.3	4.9–15	0.1–0.8	357–1985	0.5–2.4
			Cement	21–45	2.5–12	8.2–20	0.3–1.4	1088–1256	1.9–2.2
Carbon	105–263	510–5000	Lime	10–18	3.2–6.7	8–16	0.4–2.1	369–2588	0.1–2.5
			Cement	20–119	3.5–12	7–39	0.3–5.2	422–3004	0.3–2.6
Glass	35–149	520–1850	Lime	10–21	2.2–9	5–16	0.2–2.4	255–2239	0.04–3.6
			Cement	15–79	3.5–9.9	7.6–34	0.1–7.2	172–1978	0.3–2.6
PBO	191–282	2470–3910	Lime	15	2	6	0.1–0.7	1817–2572	0.4–1.2
			Cement	20–80	2–9.3	6–39	0.1–0.6	1437–4670	0.2–2.0
Steel	130–210	1100–3210	Lime	13–21	3.2–5.5	9–15	0.06–2.6	2548–3364	1.2–2.2
			Cement	22–50	2.5–11.2	10–31.5	0.7–3.8	2231–3246	1.1–2.8
			Geopolymer	50–57	8–10.4	20–22.1	0.8–0.9	2231–2951	1.1–1.8

## Data Availability

Not applicable.

## Nomenclature

*γ_m_*	Fracture surface work for forming a crack in the matrix
*ε_cs_*	Composite strain at crack stabilization stage
*ε_cu_*	Composite strain at failure
*ε_mu_*	Failure strain of the matrix
*ε_fu_*	Failure strain of the fiber
*σ_c_*_1_	Composite stress corresponding to the formation of the first crack
*σ_cdrop_*	Drop in composite stress after the formation of the first crack
*σ_cu_*	Composite tensile failure stress
*σ_mu_*	Tensile strength of the matrix
*σ_fu_*	Tensile strength of the fiber
*τ_s_*	Frictional bond strength
*a*	Factor for calculating the composite strain at the crack stabilization stage
*d_f_*	Fiber diameter
*E_c_*_1_	Initial elastic modulus of the composite
*E_c_*_2_	Elastic modulus of the composite after the formation of the first crack
*E_cxo_*	Elastic modulus of the composite along the debonded interface
*E_m_*	Elastic modulus of the matrix
*E_f_*	Elastic modulus of the fiber
*f_m,c_*	Compressive strength of the matrix
*f_m,fl_*	Flexural strength of the matrix
*G_m_*	Matrix fracture energy
*k_i_*	Equivalent stiffness of a composite zone with full fiber–matrix bond
*k_xo_*	Equivalent stiffness of the composite along the debonding length
*l_tot_*	Total length of specimen
*l_i_*	Lengths of specimen sections with full matrix–fiber bond
*x_o_*	Debonding length
*V_f_*	Fiber volume
*V_f,crit_*	Critical fiber volume
*V_m_*	Matrix volume
